# The Equilibrium Molecular Structure of Cyclic (Alkyl)(Amino) Carbene Copper(I) Chloride via Gas-Phase Electron Diffraction and Quantum Chemical Calculations

**DOI:** 10.3390/molecules28196897

**Published:** 2023-10-01

**Authors:** Alexander V. Belyakov, Ekaterina P. Altova, Anatoliy N. Rykov, Pavel Yu. Sharanov, Igor F. Shishkov, Alexander S. Romanov

**Affiliations:** 1 Saint-Petersburg State Technological Institute, 190013 Saint Petersburg, Russia; 2 Department of Chemistry, Moscow State University, 119992 Moscow, Russia; altovaek@gmail.com (E.P.A.); pashka000@mail.ru (P.Y.S.); 3 Department of Chemistry, University of Manchester, Oxford Rd., Manchester M13 9PL, UK

**Keywords:** carbene–metal–halide, phosphorescence, cyclic (alkyl)(amino) carbene, electron diffraction, computational

## Abstract

Copper-centered carbene–metal–halides (CMHs) with cyclic (alkyl)(amino) carbenes (CAACs) are bright phosphorescent emitters and key precursors in the synthesis of the highly promising class of the materials carbene–metal–amides (CMAs) operating via thermally activated delayed fluorescence (TADF). Aiming to reveal the molecular geometry for CMH phosphors in the absence of the intermolecular contacts, we report here the equilibrium molecular structure of the (CAAC)Cu(I)Cl (**1**) molecule in the gas-phase. We demonstrate that linear geometry around a copper atom shows no distortions in the ground state. The structure of complex **1** has been determined using the electron diffraction method, supported by quantum chemical calculations with RI-MP2/def2-QZVPP level of theory and compared with the crystal structure determined by X-ray diffraction analysis. Mean vibrational amplitudes, *u_ij_*_,h1_, and anharmonic vibrational corrections (*r_ij_*_,e_ • *r_ij_*_,a_) were calculated for experimental temperature T = 20 °C, using quadratic and cubic force constants, respectively. The quantum theory of atoms in molecules (QTAIM) and natural bond order (NBO) analysis of wave function at MN15/def2TZVP level of theory revealed two Cu^…^H, three H^…^H, and one three-center H^…^H^…^H bond paths with bond critical points. NBO analysis also revealed three-center, four-electron hyperbonds, (3c4e), [π(N–C) *n*_π_(Cu) ↔ *n*_π_(N) π(N–Cu)], or [N–C: Cu ↔ N: C–Cu] and *n*_π_(Cu) → π(C–N)* hyperconjugation, that is the delocalization of the lone electron pair of Cu atom into the antibonding orbital of C–N bond.

## 1. Introduction

Organic light-emitting diode (OLED) is an emerging technology enabling commercial applications in display and lighting industries and providing products with flexible, foldable, and transparent form factors. Thanks to the self-emissive nature of the technology, OLEDs are highly energy-efficient, enable high-contrast and true black color displays, together with many other desirable characteristics. The efficiency of OLEDs strongly correlates with the type of luminophore employed in the emitting layer and harvesting of the “bright” singlet and “the dark” triplet excitons generated in a ratio 1 per 3, respectively. Therefore, fluorescent emitters have a 25% limit for the maximum internal quantum efficiency (IQE) due to exclusive harvesting of the singlet excitons while losing 75% of the “dark” triplet states in non-radiative events. To relax the spin-selection rule and harvest the remaining triplet excitons, one needs to use phosphorescent emitters containing heavy elements with strong spin–orbit coupling coefficients [1]. Phosphorescent octahedral complexes of iridium appeared to be particularly successful [2] at enabling commercial red and green PhOLEDs. However, blue PhOLED suffers from the degradation of the blue emitter, thus requiring the development of robust and bright material. Thermally activated delayed fluorescence (TADF) emitters [3] can serve as an alternative approach due to their comparable performance in phosphorescent OLED devices [4] and nearly unlimited molecular design strategies available for organic and organometallic TADF emitters. Numerous advancements in phosphorescent [5] and TADF [6] materials have been extensively reviewed while necessitating the need for the further development of materials with a greater thermodynamic stability and short (submicrosecond excited state lifetime) and minimized nonradiative pathways to enable bright and stable blue OLED. For instance, understanding the molecular vibrations and differences in molecular geometry between the ground and excited states guides the molecular design to minimize geometry distortions (nonradiative decays) for prospective luminophores.

**Figure 1:**
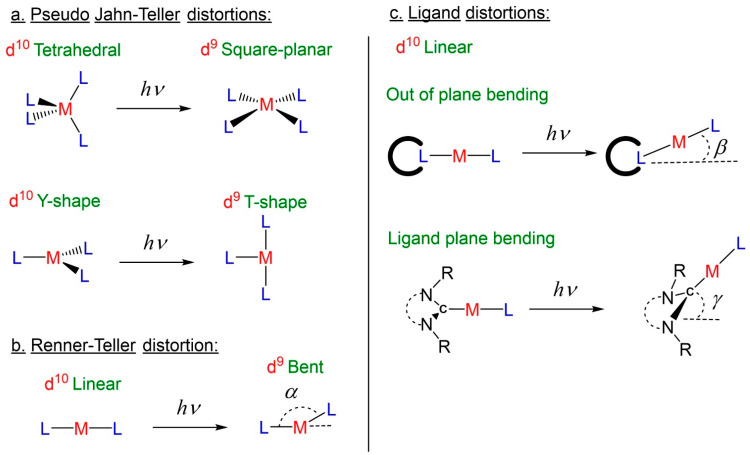
Schematic illustration of the molecular distortions reported for d^10^-coinage metal complexes emitting from ^3^MLCT or hybrid ^3^M(X)LCT excited state.

Luminescent coinage metal (copper, silver, or gold) complexes with d^10^-electron configuration attracted particular attention due to the absence of the low-energy triplet metal-centered (^3^MC) excited state (d-d transition) upon photoexcitation. This unique advantage of coinage metal complexes enables bright photoluminescence via fluorescence, TADF, or phosphorescence mechanisms [7,8], whereas classical transition metal complexes with partially filled 5d orbitals (rhenium, osmium, iridium, or platinum) emit light almost exclusively via metal-to-ligand charge transfer ^3^MLCT-phosphorescence [9]. Numerous molecular designs have been reported to achieve bright coinage metal-based luminophores thanks to a variety of coordination modes available for the metal center, as shown in Figure 1: four-coordinated (tetrahedral), three-coordinated (trigonal planar T- and Y-shape), and two-coordinated (linear) [10,11,12,13,14,15,16,17,18]. However, a photoexcited organometallic complex may experience fast nonradiative relaxation to the ground via geometric distortions, which depends on the coordination mode of the coinage metal complex (see Figure 1). For instance, tetrahedral complexes suffer from pseudo Jahn–Teller distortions, resulting in a more flattened or square planar geometry (see Figure 1a) [19]. Three-coordinated coinage metal complexes commonly experience the Y- to T-shape geometry distortion [12,20]. Two-coordinated metal complexes with linear geometry are prone to nonradiative decay associated with the bending, Figure 1b, or Renner–Teller distortion [21,22]. Such bending distortions are commonly reported for the linear CarbeneM(I)X (X = halide, pseudo-halide, or aryl) complexes emitting phosphorescence from the triplet state with ^3^MLCT or hybrid ^3^M(X)LCT character [23,24]. Therefore, to prevent such energy loss or nonradiative decay, one should avoid changing the oxidation state of the coinage metal and increase the steric protection around the metal atom [24]. Recently, carbene–metal–amides (CMAs) have emerged as a promising class of linear coinage metal complexes that show bright TADF with a submicrosecond excited state lifetime due to a ligand–metal–ligand charge transfer state, L(M)LCT [25,26,27,28,29,30,31,32]. A coinage metal atom serves as a bridge between carbene and amide ligands, where it provides minimal participation but an efficient charge transfer between the ligands. This results in highly efficient and submicrosecond TADF luminescence with up to unity photoluminescence quantum yields (PLQYs).

While linear carbene–metal–halide (CMH) complexes are key precursors to highly promising CMA materials, insights towards the photoluminescence behavior and nonradiative decays are significantly less reported. Such oversight may be due to lack of interest in generally non-emissive gold and silver CMH complexes compared with bright copper analogs. Recently we and others suggested alternative molecular distortion, such as out of plane bending or ligand bending, while the metal fragment retains its linear geometry [33]. This is revealed by comparison of the geometries of the theoretically calculated triplet excited state geometry of the CMH in the gas-phase and single-crystal molecular geometry obtained from the X-ray diffraction experiment (Figure 1c).

**Figure 2:**
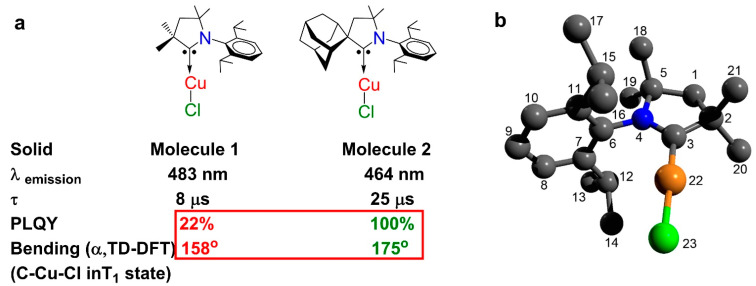
(**a**) Molecular structure for CMH molecules 1 and 2 with key photophysical parameters in the solid state and the bending distortion (angle, α) calculated by TDDFT in the gas-phase; (**b**) the gas-phase molecular structure of the molecule **1** with a numbering scheme.

The brightest linear coinage metal complexes, CMH or CMA [25,26,27,28,29,30,31,32,33,34], were first demonstrated based on cyclic (alkyl)(amino) carbenes (CAACs) [16]. In the series of the copper CMH and CMA complexes, the PLQY efficiency increases with an increase in the CAAC steric protection of the metal center in the following order methyl < ethyl < cyclohexyl < adamantyl < menthyl-substituted CAAC carbenes [19]. For instance, minimal bending vibrations can be achieved already with an adamantyl-substituted CAAC ligand (angle α is ca. 175° and PLQY 100%, Figure 2), whereas the smallest dimethyl-substituted CAAC carbene results in a decrease in the PLQY down to 22% for complex **1** (Figure 2) due to the significant bending distortion of the linear geometry (angle α is ca. 158°) in the T_1_-triplet excited state. Therefore, we select complex **1** as our target molecule due to its higher susceptibility towards bending distortion to make comparisons between experimental molecular structures in the gas and crystal phases where intermolecular interactions and lattice forces dictate the molecular geometry.

The aim of this work is to provide the first empirical insights into the CMH molecular structure in the gas-phase by performing an electron diffraction experiment. The absence of intermolecular contacts for the CMH molecule **1** under a high vacuum will reveal the impact of the intramolecular interactions on its molecular geometry, supported by theoretical calculations. Therefore, the similarities and differences between gas-phase and crystal geometries based on the model molecule **1** will facilitate developments in the design of future materials to produce and explain efficient light-emitting materials.

## 2. Results and Discussion

Dimethyl cyclic (alkyl)(amino) carbenes (CAACs) copper(I) chloride (molecule **1**, Figure 2) was obtained as previously reported by us and others [16,34]. Complex **1** was sublimed twice on a gram scale at 160 °C and 1 × 10^–6^ mbar pressure to obtain high-purity samples suitable for the gas-phase electron diffraction experiment. This molecule has been thoroughly studied both experimentally and computationally with key photophysical data collated in Figure 2. Complex **1** emits blue–white phosphorescence with peak max at 483 nm and PLQY up to 22% in the crystalline solid (Figure 2a).

The electron diffraction experiment and equipment are described in the Supplementary Materials. The Cartesian coordinates of atoms were calculated according to an algorithm given in the literature [35]. For the ring closure, the calculation of the coordinates is not terminated at the last atom in the ring but rather continued for the three dummy atoms according to the algorithm rules [35]. The problem of ring closure is reduced to the iterative solution of nonlinear equations with respect to the dependent geometrical parameters so that the Cartesian coordinates of dummy atoms coincide with those of the first three atoms of the ring.

When refining structural parameters, the minimized functional has the following form:

(1) $$Q=\sum_{s}ws{∆s}^{2}=\sum_{s}{ws\left[{sM}^{obs}\left(s\right)-k\cdot {sM}^{calc}\;\left(s\right)\right]}^{2}\;$$

where *s* = (4π/λ) sin(θ/2) is the parameter of the scattering angle θ; λ is the wavelength of the electron beam; *w*_s_ is a weight function; *sM(s)* is the molecular intensity function; and *k* is the scale factor. As a criterion of the minimum of the functional, the value of the *R*-factor was taken:

(2) $$R={\left(Q/\sum_{s}{ws\left[{sM}^{obs}\left(s\right)\right]}^{2}\right)}^{1/2}$$

Least-squares structure refinements were carried out using a modified version of the KCED25 program [36]. Weight matrices were diagonal. Short-distance data were taken with weights of 0.5, and long-distance ones were taken with unity weights.

**Figure 3:**
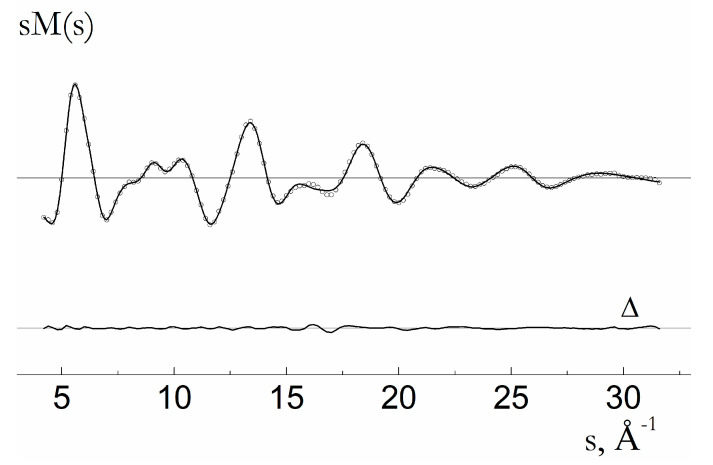
Molecular intensity curves of molecule **1** (experimental and calculated curves are shown with dotted and solid lines, respectively). The difference curve (Δ) is obtained by subtracting theoretical values from the experimental ones.

**Figure 4:**
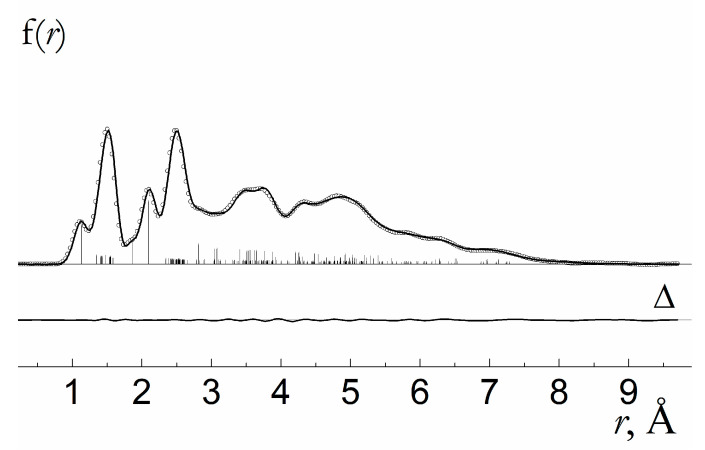
Radial distribution curves of molecule **1** (experimental and calculated curves are shown with dotted and solid lines, respectively). The difference curve (Δ) is obtained by subtracting theoretical values from the experimental ones.

The molecular structure of **1,** as shown in Figure 2, was specified by 55 bond lengths, 57 bond angles, and 57 dihedral angles. Among them, two bond lengths, six bond angles, and eight dihedral angles were the ring closure parameters (Table 1). Geometrical parameters and vibrational amplitudes were refined in groups with constant differences obtained from theoretical MP2 and DFT estimates, respectively. Particularly, the root mean square amplitudes were refined in seven groups according to the specific ranges of the radial distribution curve (Figure 3 and Figure 4): 1.0−1.2; 1.2−1.7; 1.7−2.3; 2.3−2.7; 2.7–4.0; 4.0−5.6; and 5.6−9.5. The final sM(s) molecular intensity and f(r) radial distribution curves are shown in Figure 3 and Figure 4, respectively. Correlation coefficients larger 70% are observed for the following refined parameters: R2(C1-C2)/R12(C12-C7) − 82; u(1.7−2.3)/R23(Cl23-Cu22) − 85; Scale(1)/u(1.2−1.7) − 77. The best correspondence between the experimental and calculated molecular intensities was obtained for the final set of geometrical parameters listed in Table 1.

### 2.1. Comparison with the Single-Crystal X-ray Diffraction Data

**Figure 5:**
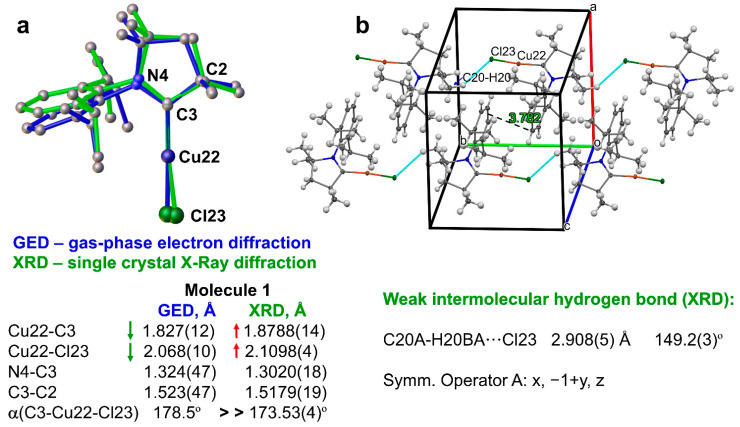
(**a**) Superposition of the gas-phase electron diffraction (GED) and single-crystal X-ray diffraction (XRD) geometries for complex **1** (overlay via Cu22, C3, and N4 atoms), demonstrating a distortion of the linear geometry around copper center and showing key geometrical parameters. (**b**) Packing diagram for **1** with intermolecular contacts between neighboring molecules shown as a dashed line.

We compare the molecular structure of the **1** complex from the gas-phase electron diffraction with one from the single-crystal X-ray diffraction experiment to reveal the role of the intermolecular contacts on the key geometrical parameters (bond lengths and angles) and linear geometry bending distortions of **1**. Single crystals for the X-ray diffraction study of **1** were obtained by slow evaporation of the CH_2_Cl_2_ solution. The structure of **1** has previously been reported and studied [16,34]; however, no lower precision has been obtained than that in the present work or containing the crystallized solvent molecules in the unit cell that would alter and mask the role of the intermolecular interactions between neighboring molecules of **1**. (Figure 1). The title compound crystallizes with one independent molecule in the monoclinic space group *P2_1_/n*. The crystal structure (Figure 5) shows a linear geometry, without any close copper–copper contacts. The Cu–C_carbene_ and Cu–chloride bond lengths of 1.8788(14) and 2.1098(4) Å are longer by 0.04 Å compared to the distances observed in the gas-phase. Moreover, the crystal linear geometry around copper demonstrates a significant bending distortion with deviations up to 6.5(4)° from the ideal angle of 180° and the geometry of **1** in the gas-phase (Figure 5a). Such longer covalent bonds around copper atom together with significant bending distortion are likely to be associated with the intermolecular contacts that are present in the crystal but absent for the nearly isolated molecules of **1** in the gas-phase. Analysis of the intermolecular contacts shows that molecules of **1** form a three-dimensional network via multiple weak C-H(carbene)···π(aryl) and π(aryl)-π(aryl) stacking interactions 3.78(1) Å contacts for the latter (Figure 5b). Within this 3D network, we can distinguish head-to-tail chains enabled by weak intermolecular hydrogen bond C20A-H20BA···Cl23, where the D-A = 2.908 (5) Å, D-A angle is 149.2(3)° and A is a symmetry operator x; –1+y; z, blue dashed line in Figure 5b. The neighboring chains of molecules **1** have alternating and antiparallel orientation, as shown in Figure 5b. Multiple weak C-H(carbene)···π(aryl) and π(aryl)-π(aryl) stacking interactions connect all antiparallel chains into the 3D network of the crystal of **1**. Therefore, it is primarily the hydrogen bond C20A-H20BA···Cl23 and weak intermolecular interactions which originate from the distortion of the linear geometry around Cu22 atom in the crystalline environment and affect the photophysical properties of complex **1** in the solid state. 

### 2.2. Analysis of the Intramolecular Contacts in the Gas-Phase with the Quantum Theory of Atoms in Molecules (QTAIM)

**Figure 6:**
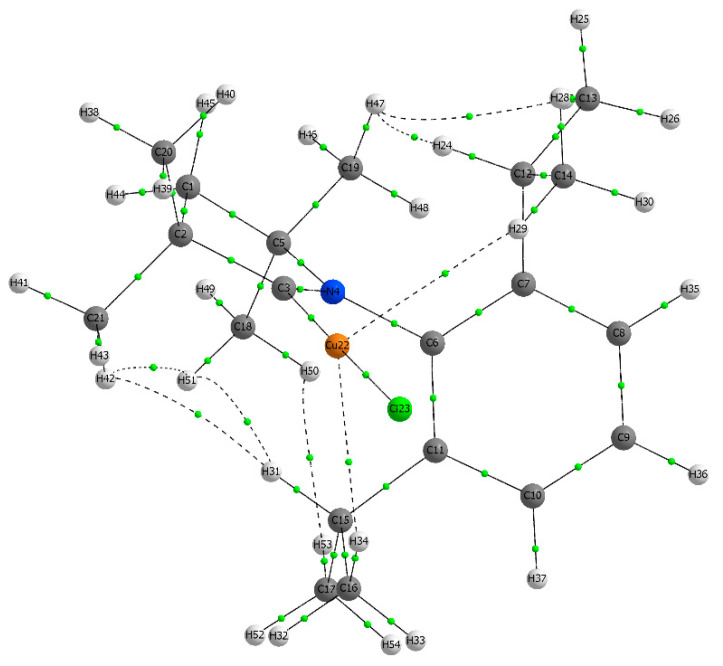
Molecular graph of molecule 1 from QTAIM analysis of wave function.

Next, we consider the structure of complex **1** in the gas-phase to analyze the intramolecular contacts with the help of the QTAIM analysis of the wave function at MN15/def2TZVP level of theory. We revealed two Cu^…^H, three H^…^H, and one three-center H^…^H^…^H bond paths with bond critical points, as shown in Figure 6. Previously, various intramolecular contacts determined using an X-ray diffraction experiment were used to claim and explain the unique photophysical behavior of the organic or organometallic luminophores [37,38,39]. However, it is only the gas-phase structural characterization that may provide an explicit experimental confirmation for the intramolecular contact of interest that favors a particular geometry in the absence of the intermolecular contacts.

**Figure 7:**
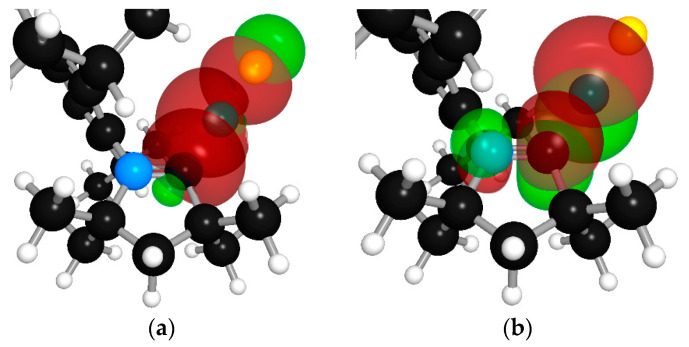

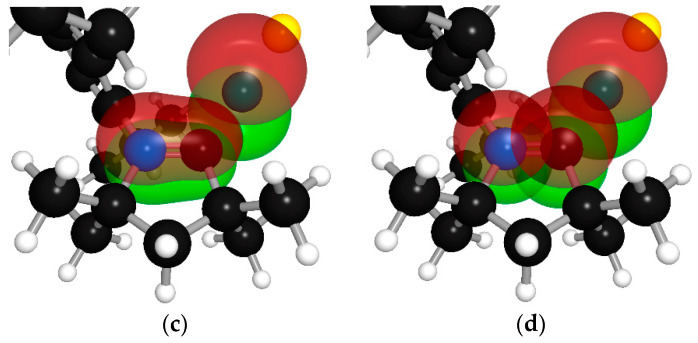
Surface views of overlapping orbitals (**a**) n_σ_(C) → n(Cu)* ← *n*_σ_(Cl); corresponding values of Δ*E_n_*_→n*_ donor–acceptor stabilization energy are 143.0 and 77.0 kcal mol^–1^, respectively. (**b**) *n*_π_(Cu) → π(C–N)*, that is delocalization of lone electron pair of Cu atom into antibonding orbital of C–N bond, Δ*E_n_*_→π*_ is 114.9 kcal mol^–1^. (**c**) 3c4e hyperbond [π(N–C) *n*_π_(Cu) ↔ *n*_π_(N) π(N–Cu)] or [N–C: Cu ↔ N: C–Cu]. (**d**) Three NHO on N, C, and Cu atoms corresponding to 3c4e hyperbond.

According to NBO analysis, the Cu–C_carbene_ and Cu–chloride bonds are formed by the delocalization of a bonding lone pair of carbon and chlorine atoms into the vacant orbital of a copper atom [n_σ_(C) → n(Cu)* ← *n*_σ_(Cl)]. NBO analysis also revealed three-center, four-electron hyperbonds, (3c4e), [π(N–C) n_π_(Cu) ↔ n_π_(N) π(N–Cu)] or [N–C: Cu ↔ N: C–Cu], and n_π_(Cu) → π(C–N)* hyperconjugation, that is the delocalization of the lone electron pair of Cu atom into the antibonding orbital of C–N bond of the CAAC-carbene. Surface views of overlapping orbitals are shown in Figure 7.

## 3. Materials and Methods

### 3.1. Computational Details

The geometry optimization runs were performed at the all-electron second order Møller–Plesset perturbation theory level with the resolution-of-identity technique (RI-MP2) [40] and the use of the def2-QZVPP basis sets [41], and at the MN15/def2TZVP level of theory (DFT) [42]. The calculations were carried out with the Orca 5.0.1 [43] and Gaussian16 (Revision C01) [44] program packages, respectively. In the case of DFT simulations, normal coordinate analysis was used to prove the character of the stationary point found on the potential energy surface. A summary of the residual results can be found in Table 1 and the Supplementary Materials Table S2.

Mean amplitudes (*u*_ij,h1_) and vibrational corrections (*r*_ij,e_ − *r*_ij,a_) necessary for the gas-phase electron diffraction analysis (GED) were computed using quadratic and cubic force fields, respectively, at the first-order perturbation theory level, taking into account curvilinear kinematic effects as implemented in the SHRINK computer program [45,46,47]. Quadratic and cubic force fields were calculated at the DFT level with an MN15 functional [42] and def2-QZVPP basis sets [41].

NBO analysis of the DFT wave function [48,49] was performed using the NBO 7.0 computer program [50]. Topological analyses of electron density were performed with the use of the QTAIM method [51]. The AIMAll computer program [52] and MN15/def2TZVP level of theory were used.

### 3.2. X-ray Diffraction Experiments

The crystal of **1** was mounted in oil on a MiTeGen loop and fixed on the diffractometer in a cold nitrogen stream. Data were collected using a dual wavelength Rigaku FR-X rotating anode diffractometer via CuKα (λ = 1.54146 Å) radiation, equipped with an AFC-11 4-circle kappa goniometer, VariMAXTM microfocus optics, a Hypix-6000HE detector, and an Oxford Cryosystems 800 plus nitrogen flow gas system, at a temperature of 100 K. Data were collected and reduced using CrysAlisPro v42 [53,54]. Absorption correction was performed using empirical methods (SCALE3 ABSPACK) based upon symmetry-equivalent reflections combined with measurements at different azimuthal angles.

Structures were solved by direct method/intrinsic phasing and refined by the full-matrix least-squares against F^2^. All non-hydrogen atoms were refined with anisotropic atomic displacement parameters. All hydrogen atoms were positioned geometrically and constrained to ride on their parent atoms with C-H = 0.95–1.00 Å and *U_iso_* = 1.2−1.5 *U_eq_* (parent atom). All calculations were performed using the SHELXL software and Olex2 graphical user interface [54,55].

**Crystal Data** for **1**. CCDC number: 2298540. C_20_H_31_ClCuN (*M* = 384.45 g/mol): monoclinic, space group P2_1_/n (no. 14), *a* = 10.6731(2) Å, *b* = 10.0300(2) Å, *c* = 18.8924(3) Å, *β* = 90.144(2)°, *V* = 2022.45(6) Å^3^, *Z* = 4, *T* = 100.00(12) K, μ(Cu Kα) = 2.710 mm^−1^, *Dcalc* = 1.263 g/cm^3^, 11,228 reflections measured (9.362° ≤ 2Θ ≤ 151.4°), 4004 unique (*R*_int_ = 0.0212, R_sigma_ = 0.0254) which were used in all calculations. The final *R*_1_ was 0.0285 (I > 2σ(I)) and *wR*_2_ was 0.0804 (all data).

### 3.3. Gas-Electron Diffraction Experiments

The electron diffraction patterns were recorded in Moscow State University on the EG-100M apparatus using the R3 sector made of brass. The electron wavelength was calibrated against gaseous CCl_4_. The structural parameters of the CCl4 molecule were taken from [56]. Information about the experimental conditions for all datasets used in the present investigation is given in Table S1. Photo films (TASMA FT-41P) were scanned with the use of the Epson Perfection Photo 4870 commercial scanner in the 16-bit/4800-dpi gray scale scanning mode and with the use of the VueScan computer program [57]. This program enables one to retrieve data directly from the detector without any modifications. The data were processed using a computer program written by A.V.B. as in [58]. Preliminarily, the high resolution was reduced by averaging over square regions of pixels, as described in [59]. With this method, mean transmittances and standard deviations were collected. The latter were used as weights for smoothing the transmittance surface with the use of 2D cubic splines [60]. The calibration of the scanner was carried out against an MD100 microdensitometer with the use of a 24 bit gray scale optical wedge of an IT8 transmissive target on Kodak Ektachrome Professional E100G film [61]. Displacements of the scanner were corrected against a special ruler manufactured by LOMO. After refinement of the center of an electron diffraction pattern using the least-squares method, the data of the scanning were transformed into the total intensity curve, taking into account 2D background. The atomic scattering factors were taken from [62]. We attempted to refine the C-Cu-Cl bond angle, resulting in a value of 177.9° which corresponds to near linear geometry around copper atom. However, we had a large uncertainty 17.3° or one sigma LSQ method. Therefore, the C-Cu-Cl bond angle was fixed on a theoretical value because the R-factor was sufficiently low –4.7%. The highest possible level of theory (MP2/def2-QZVPP) and experimental data are in good agreement; however, it is challenging to predict the C-Cu-Cl bond angle a priori.

## 4. Conclusions

We have determined the molecular structure of molecule **1** in both gas and solid states using electron and X-ray diffraction methods (GED and XRD). A comparison of the geometrical parameters between these molecular structures of molecule **1** revealed a significant bending distortion in the solid state. In contrast, in the gas-phase, molecule **1** possesses a nearly perfect linear geometry around the copper atom. We demonstrated that various intermolecular contacts such as weak C-H(carbene)···π(aryl), π(aryl)-π(aryl) stacking interactions and intermolecular hydrogen bond C20A-H20BA···Cl23 originate deviations in the linear geometry of **1** in the solid state, thus rationalizing the mediocre luminescence behavior of **1**.

Our work is a proof-of-principle demonstration that the gas-phase structural characterization of the CMH derivatives can be achieved in the presence of the heavy copper atom, commonly associated with multiple scattering problems. This fact opens a bright future for the electron diffraction method to characterize even more challenging advanced materials, which are highly sought after within and beyond the optoelectronics area. For instance, research on heavier copper analogues, such as silver and gold-based CMH and CMA materials, is underway in our laboratories. We demonstrate that the GED method and the quantum theory of atoms in molecules (QTAIM) can reveal various weak intramolecular bond paths. For instance, we found that molecule **1** possesses Cu^…^H, H^…^H, and three-center H^…^H^…^H that are likely to dictate the molecular geometry thanks to the absence of the intermolecular contacts that dominate the solid state and mask weak interactions. Our current and future findings will contribute to the molecular design prediction of bright and stable luminophores by providing experimental knowledge about the luminophore geometry in the gas-phase, which is highly important during the thermal vapor deposition OLED fabrication process.

## Figures and Tables

**Table 1:** Main equilibrium structural parameters for molecule 1 from gas-phase electron diffraction analysis (GED) and theoretical calculations at MP2 level of theory.

Parameters ^a^	GED	MP2 ^b^	Parameters	GED	MP2
R2(C1-C2)	1.555(47)	1.533	A12(C12,C7,C6)	123.3(3.3)	122.7
R3(C2-C3)	1.523(47)	1.501	A13(C13,C12,C7)	112.7	112.7
R4(N4-C3)	1.324(47)	1.302	A14(C14,C12,C7)	109.1	109.1
R5(C5-N4)	1.520(47)	1.498	A15(C15,C11,C6)	123.0	123.0
R1(C5-C1) ^c^	1.547(47)	1.525	A16(C16,C15,C11)	109.2	109.2
R6(C6-N4)	1.452(47)	1.430	A17(C17,C15,C11)	112.9	112.9
R7(C7-C6)	1.401(9)	1.398	A18(C18,C5,C1)	113.5	113.5
R8(C8-C7)	1.392(9)	1.390	A19(C19,C5,C1)	113.0	113.0
R9(C9-C8)	1.385(9)	1.383	A20(C20,C2,C3)	110.3	110.3
R10(C10-C9)	1.385(9)	1.383	A21(C21,C2,C3)	107.4	107.4
R11(C11-C10)	1.393(9)	1.391	A22(Cu22,C3,C2)	129.1(2.5)	128.4
R(C11-C6) ^c^	1.401(9)	1.399	A(23,22,3)	178.5	178.5
R12(C12-C7)	1.501(38)	1.503	D4(N4,C3,C2,C1)	−11.5	−11.5
R13(C13-C12)	1.517(38)	1.519	D5(C5,N4,C3,C2) ^c^	0.2	0.1
R14(C14-C12)	1.521(38)	1.523	D1(C1,C5,N4,C3) ^c^	11.1	11.3
R15(C15-C11)	1.501(38)	1.503	D2(C2,C1,C5,N4) ^c^	−17.2	−17.3
R16(C16-C15)	1.521(38)	1.523	D3(C3,C2,C1,C5) ^c^	18.2	18.2
R17(C17-C15)	1.517(38)	1.519	D6(C6,N4,C3,C2)	−178.0	−178.0
R18(C18-C5)	1.513(38)	1.515	D7(C7,C6,N4,C5)	90.7(10.7)	90.4
R19(C19-C5)	1.510(38)	1.512	D8(C8,C7,C6,C4)	−176.5	−176.5
R20(C20-C2)	1.514(38)	1.516	D9(9,8,7,6)	−1.1	−1.1
R21(C21-C2)	1.522(38)	1.523	D10(10,9,8,7)	−2.9	−2.9
R22(Cu22-C3)	1.827(12)	1.797	D11(11,10,9,8) ^c^	2.6	2.7
R23(Cl23-Cu22)	2.068(10)	2.052	D(6,11,10,9) ^c^	1.6	1.5
R24(C*sp*_3_-H)_av_	1.094(132)	1.086	D(7,6,11,10) ^c^	−5.8	−5.7
A3(3,2,1) ^c^	104.4(1.1)	104.4	D(8,7,6,11) ^c^	5.6	5.5
A4(4,3,2)	108.4(1.1)	108.4	D12(12,7,6,4)	10.1	10.1
A5(5,4,3) ^c^	116.7(1.1)	116.8	D13(13,12,7,8)	54.3	54.3
A1(1,5,4) ^c^	100.4(1.1)	100.4	D14(14,12,7,13)	−123.1	−123.1
A2(2,1,5) ^c^	106.7(1.1)	106.6	D15(15,11,6,4)	−10.5	−10.5
A6(6,4,3)	121.5(3.2)	121.8	D16(16,15,11,10)	66.9	66.9
A7(7,6,4)	115.4(1.8)	118.2	D17(17,15,11,16)	−122.8	−122.9
A8(8,7,6) ^c^	117.4	117.4	D18(18,5,1,2)	99.7	99.7
A9(9,8,7)	121.2	121.2	D19(19,5,1,18)	124.7	124.7
A10(10,9,8)	119.9	119.9	D20(20,2,3,4)	−132.3	−132.3
A11(11,10,9) ^c^	121.3	121.3	D21(21,2,3,20)	−119.3	−119.3
A(6,11,10) ^c^	117.3	117.3	D22(22,3,2,1)	169.2	169.2
A(7,6,11) ^c^	122.6	122.6	D(23,22,3,2)	−157.5	−157.4

^a^ R-factor = 4.7% was calculated from Equation (2), where av stands for averaged value. Parenthesized values are 3σ of LSQ method. Not refined parameters were fixed on theoretical values; see Figure 2 for the numbering scheme. ^b^ RI-MP2/def2-QZVPP level of theory. ^c^ Ring closure parameters.

## Data Availability

Details of the gas-phase diffraction experiment are available as supplementary material.

## Supplementary Materials

The following supporting information can be downloaded at: https://www.mdpi.com/article/10.3390/molecules28196897/s1, and contains the details of the gas-phase electron diffraction experiment and equipment used to collect the data.

## Sample Availability

Samples of the complex **1** are available from the authors upon reasonable request.
