# Supplementary material for: The Equilibrium Molecular Structure of Cyclic (Alkyl)(Amino) Carbene Copper(I) Chloride via Gas-Phase Electron Diffraction and Quantum Chemical Calculations
Source: Molecules. 2023 Oct 1;28(19):6897. doi: 10.3390/molecules28196897 (PMC10574683; doi:10.3390/molecules28196897)
Supplement: Supplementary file 1 [file molecules-28-06897-s001.zip › molecules-2615398-supplementary.pdf]

Supporting Information for

The equilibrium molecular structure of Cyclic (Alkyl)(amino)carbene Copper(I) Chloride by gas-phase electron diffraction and quantum-chemical calculations

Alexander V. Belyakov, Ekaterina P. Altova, Anatoliy. N. Rykov, Pavel Yu. Sharanov, Igor F. Shishkov and Alexander S. Romanov

S1. Gas-electron diffraction experiment

Table S1. Experimental conditions of the gas-phase electron diffraction experiment for **1**

	**1**	
	LD	SD
Camera distance (mm)	362.3	193.9
Nozzle temperature (K)	492	498
Accelerating voltage (kV)	60	60
Vacuum (mm Hg)	$4.0 \cdot 10^{-5}$	$3.0 \cdot 10^{-5}$
Electron beam current (μA)	3.0	3.0
Electron wavelength (Å)	0.0496975	0.0499395
Number of films used	3	1
Range of s value ($Å^{-1}$)[a]	4.2 – 16.0	13.0 – 31.6

[a] $s = (4\pi/\lambda)\sin(\theta/2)$, where θ is the scattering angle and λ is the electron wavelength. Experimental intensity curves were digitized with a step of 0.2 $Å^{-1}$.

S2. Quantum chemical calculations and GED data

Table S2. Cartesian coordinates of (**1**) molecule, (r_e, Å) optimized at the RI-MP2/def2-QZVPP and MN15/def2TZVP, (DFT) levels of theory.

Table S3. Cartesian coordinates of (**1**) molecule, (r_e, Å) by gas-phase electron diffraction, (GED).

Table S4. Total corrections $\Delta(r_{ij,e} - r_{ij,a})$ to internuclear distances $r_{ij,a}$, theoretical $u_{ij,h1}$ and experimental $u_{ij,exp}$ rms vibrational amplitudes (Å) for (**1)** molecule.

Table S5. Natural Bond Order: (total/covalent/ionic)

Table S6. Total scattering intensities and background lines for (1) molecule

Table S2. Cartesian coordinates of (**1**) molecule, (r_e, Å) optimized at the RI-MP2/def2-QZVPP and MN15/def2TZVP, (DFT) levels of theory.

	MP2			DFT		
At	*x*	*y*	*z*	*x*	*y*	*z*
C1	0.09440	−3.20878	−0.30523	0.98535	3.00001	−0.54240
C2	−1.20380	−2.43958	−0.03498	1.98894	1.85282	−0.32856
C3	−0.79139	−0.99609	−0.04690	1.12911	0.62302	−0.20018
N4	0.50887	−0.93990	−0.02994	−0.11441	0.98985	−0.07060
C5	1.24038	−2.24745	−0.00654	−0.38314	2.46745	−0.09740
C6	1.20975	0.30638	0.00665	−1.17665	0.03367	0.13285
C7	1.57761	0.89468	−1.20753	−1.84569	−0.47505	−0.98845
C8	2.32305	2.06642	−1.15099	−2.92000	−1.33277	−0.76882
C9	2.66640	2.63971	0.06017	−3.29685	−1.69501	0.51440
C10	2.21584	2.07787	1.24071	−2.56174	−1.24886	1.60003
C11	1.46387	0.90798	1.24363	−1.47647	−0.39111	1.43451
C12	1.08193	0.39483	−2.53497	−1.36664	−0.24859	−2.40807
C13	2.19602	0.23584	−3.55456	−2.48167	0.22186	−3.33916
C14	0.01250	1.34488	−3.05781	−0.74665	−1.54473	−2.93746
C15	0.84438	0.43394	2.52780	−0.59961	−0.08578	2.63266
C16	−0.23296	1.42096	2.95837	0.13219	−1.36306	3.05538
C17	1.86083	0.25086	3.64135	−1.38098	0.47799	3.81743
C18	1.86814	−2.45921	1.35538	−0.79881	2.95089	1.28625
C19	2.32642	−2.28003	−1.05766	−1.49099	2.80993	−1.08211
C20	−2.25366	−2.69548	−1.09856	2.96171	1.69447	−1.49165
C21	−1.78989	−2.73513	1.33982	2.77403	1.98140	0.98074
Cu22	−1.83835	0.46390	−0.06155	1.69902	−1.19019	−0.18593
Cl23	−2.99318	2.15969	−0.09916	2.28543	−3.20821	−0.18316
H24	0.60852	−0.57110	−2.38332	−0.57898	0.50841	−2.39644
H25	1.80297	−0.18904	−4.47421	−2.07657	0.44827	−4.32725
H26	2.63367	1.19808	−3.80445	−3.23380	−0.55943	−3.46784
H27	2.99200	−0.40706	−3.19182	−2.98582	1.11201	−2.95877
H28	−0.39854	0.97285	−3.99297	−0.34761	−1.39312	−3.94243
H29	−0.79929	1.46150	−2.34537	0.05999	−1.90067	−2.29134
H30	0.43739	2.32859	−3.24179	−1.50420	−2.33097	−2.98742
H31	0.35632	−0.51722	2.34146	0.15772	0.64226	2.33542
H32	−0.73876	1.06008	3.85044	0.81658	−1.15244	3.87967
H33	0.20866	2.38760	3.18804	−0.58629	−2.11360	3.39468
H34	−0.97417	1.57073	2.17861	0.70327	−1.79788	2.23129
H35	2.61858	2.54887	−2.07001	−3.45012	−1.74246	−1.62061
H36	3.25013	3.54657	0.08218	−4.13679	−2.36119	0.66524
H37	2.42753	2.56987	2.17768	−2.81218	−1.59354	2.59651
H38	−2.50922	−3.75255	−1.12068	3.57436	2.59328	−1.59544
H39	−3.15422	−2.12486	−0.89236	3.61948	0.83927	−1.32165
H40	−1.89018	−2.40858	−2.08164	2.42665	1.53047	−2.43006

(to be continued)

Table S2. *continued*

	MP2			DFT		
At	*x*	*y*	*z*	*x*	*y*	*z*
H41	−2.11698	−3.77158	1.37967	3.42653	2.85625	0.93304
H42	−1.06449	−2.57550	2.13260	2.11046	2.09261	1.84139
H43	−2.64428	−2.09319	1.53203	3.38932	1.09347	1.14032
H44	0.13306	−3.49833	−1.35304	1.25980	3.90104	0.00971
H45	0.17150	−4.11288	0.29237	0.94637	3.25820	−1.60421
H46	2.82885	−3.24170	−1.00217	−1.65442	3.88871	−1.05993
H47	1.92142	−2.16844	−2.05649	−1.22545	2.52889	−2.10082
H48	3.06428	−1.50081	−0.88531	−2.42706	2.31732	−0.80912
H49	2.41003	−3.40062	1.34489	−1.01266	4.01961	1.23626
H50	2.57509	−1.66498	1.57575	−1.70622	2.43718	1.61012
H51	1.12827	−2.50095	2.14637	−0.01849	2.79293	2.03081
H52	1.37324	−0.14208	4.52942	−0.69724	0.72971	4.63032
H53	2.66019	−0.42844	3.36310	−1.94742	1.37274	3.55526
H54	2.31218	1.20080	3.91342	−2.08498	−0.26258	4.20317

Table S3. Cartesian coordinates of (**1**) molecule, (r_e, Å) by gas−phase electron diffraction, (GED).

		GED					
At	*x*	*y*	*z*	At	*x*	*y*	*z*
C1	0.00000	0.00000	0.00000	H28	−1.89742	3.43697	−3.89435
C2	−1.55496	0.00000	0.00000	H29	−2.77241	3.63666	−2.35999
C3	−1.93272	1.47568	0.00000	H30	−1.99331	5.03068	−3.12147
N4	−0.84409	2.18529	0.25133	H31	−1.60931	2.47681	2.59835
C5	0.44552	1.40798	0.46165	H32	−3.58677	3.28437	3.79321
C6	−0.88991	3.63279	0.35946	H33	−3.32498	4.92274	3.16752
C7	−0.66211	4.33174	−0.83263	H34	−3.75234	3.61469	2.05531
C8	−0.62166	5.72134	−0.76305	H35	−0.45350	6.29031	−1.66453
C9	−0.82285	6.38615	0.43557	H36	−0.78148	7.46354	0.46909
C10	−1.12847	5.66836	1.57987	H37	−1.35443	6.19652	2.49347
C11	−1.18635	4.27689	1.56760	H38	−1.80769	−1.73660	−1.24340
C12	−0.60488	3.66857	−2.17785	H39	−3.22106	−0.65764	−1.20386
C13	0.59566	4.10571	−2.99551	H40	−1.78826	−0.20294	−2.14121
C14	−1.89513	3.95678	−2.93053	H41	−1.90735	−1.69046	1.28327
C15	−1.69913	3.55148	2.77724	H42	−1.74780	−0.15896	2.16654
C16	−3.17875	3.85837	2.95464	H43	−3.23242	−0.50386	1.26573
C17	−0.93786	3.90197	4.04184	H44	0.36539	−0.17612	−1.01792
C18	0.84415	1.46769	1.91976	H45	0.41661	−0.78007	0.64480
C19	1.56630	1.97523	−0.37629	H46	2.46980	1.38686	−0.18889
C20	−2.12779	−0.68898	−1.22101	H47	1.34080	1.92079	−1.44291
C21	−2.14502	−0.62129	1.25765	H48	1.77403	3.01618	−0.10798
Cu22	−3.56706	2.24746	−0.26655	H49	1.78289	0.92021	2.04797
Cl23	−5.39186	3.16706	−0.58262	H50	1.00942	2.50370	2.22945
H24	−0.55082	2.58598	−2.02792	H51	0.09304	1.01833	2.57248
H25	0.63261	3.54883	−3.93708	H52	−1.31354	3.30931	4.88201
H26	0.53061	5.16831	−3.24818	H53	0.13555	3.71834	3.94546
H27	1.53651	3.94563	−2.46161	H54	−1.07481	4.95657	4.29968

Table S4. Total corrections $\Delta(r_{ij,e} - r_{ij,a})$ to internuclear distances $r_{ij,a}$, theoretical $u_{ij,h1}$ and experimental $u_{ij,exp}$ rms vibrational amplitudes (Å) for molecule (**1**)

Type	*At.Num.*	$r_{ij,a}$	$r_{ij,e} - r_{ij,a}$[a]	$u_{ij,exp}$	$u_{ij,h1}$[b]
C C	1- 2	1.567	-0.0123	0.054	0.054
C C	2- 3	1.532	-0.0083	0.053	0.053
N C	3- 4	1.330	-0.0060	0.042	0.042
N C	4- 5	1.536	-0.0155	0.055	0.055
C C	1- 5	1.557	-0.0096	0.054	0.053
N C	4- 6	1.462	-0.0095	0.049	0.049
C C	6- 7	1.411	-0.0101	0.047	0.047
C C	7- 8	1.399	-0.0070	0.047	0.046
C C	8- 9	1.392	-0.0063	0.046	0.045
C C	9-10	1.391	-0.0061	0.046	0.045
C C	10-11	1.400	-0.0070	0.047	0.046
C C	6-11	1.408	-0.0076	0.047	0.047
C C	7-12	1.514	-0.0130	0.053	0.052
C C	12-13	1.532	-0.0152	0.053	0.053
C C	12-14	1.534	-0.0123	0.054	0.053
C C	11-15	1.512	-0.0112	0.053	0.052
C C	15-16	1.534	-0.0125	0.054	0.053
C C	15-17	1.532	-0.0147	0.053	0.053
C C	5-18	1.525	-0.0120	0.053	0.053
C C	5-19	1.521	-0.0108	0.053	0.052
C C	2-20	1.530	-0.0153	0.053	0.053
C C	2-21	1.534	-0.0123	0.054	0.053
CuC	3-22	1.848	-0.0205	0.056	0.063
CuCl	22-23	2.078	-0.0098	0.060	0.057
C H	12-24	1.111	-0.0168	0.079	0.076
CuN	4-22	2.794	-0.0216	0.098	0.098
CuC	2-22	3.031	-0.0027	0.111	0.110
CuC	6-22	3.062	0.0167	0.184	0.183
CuC	20-22	3.389	0.0181	0.201	0.201
CuC	21-22	3.520	0.0259	0.196	0.196
CuC	16-22	3.629	-0.0070	0.273	0.273
CuC	11-22	3.601	0.0257	0.252	0.252
CuC	7-22	3.538	0.0818	0.256	0.256
CuC	14-22	3.490	0.0895	0.294	0.294
CuC	15-22	3.854	-0.0525	0.268	0.268
CuC	12-22	3.747	0.0537	0.271	0.271
CuC	5-22	4.190	-0.0268	0.106	0.088
CuC	1-22	4.240	-0.0153	0.109	0.092
CuC	10-22	4.515	0.0737	0.323	0.306
CuC	8-22	4.465	0.1164	0.328	0.310
CuC	18-22	5.010	-0.0249	0.168	0.150
CuC	9-22	4.905	0.1101	0.339	0.322
CuC	19-22	5.180	-0.0378	0.165	0.147
CuC	17-22	5.377	-0.0651	0.294	0.276
CuC	13-22	5.268	0.0453	0.293	0.275
ClN	4-23	4.753	-0.0267	0.245	0.227
ClC	3-23	3.903	-0.0091	0.089	0.089
ClC	16-23	4.251	-0.0216	0.352	0.682

(to be contined)

Table S4. *continued*

Type	*At.Num.*	$r_{ij,a}$	$r_{ij,e} - r_{ij,a}$ [a]	$u_{ij,exp}$	$u_{ij,h1}$ [b]
ClC	14-23	4.284	0.0013	0.352	0.767
ClC	11-23	4.833	0.0187	0.352	0.558
ClC	7-23	4.838	0.0392	0.352	0.610
ClC	2-23	4.965	0.0440	0.209	0.191
ClC	15-23	5.043	-0.0355	0.352	0.572
ClC	20-23	5.011	0.0817	0.352	0.396
ClC	12-23	5.070	0.0010	0.352	0.646
ClC	21-23	5.208	0.1095	0.352	0.401
ClC	10-23	5.313	0.0827	0.352	0.712
ClC	8-23	5.314	0.0996	0.352	0.755
ClC	9-23	5.564	0.1171	0.345	0.778
ClC	5-23	6.203	-0.0178	0.204	0.182
ClC	1-23	6.260	0.0202	0.145	0.124
ClC	17-23	6.485	-0.0228	0.345	0.603
ClC	13-23	6.511	0.0120	0.345	0.682
ClC	18-23	6.944	-0.0132	0.292	0.271
ClC	19-23	7.108	-0.0450	0.341	0.320
N C	2- 4	2.327	-0.0148	0.053	0.056
N C	1- 4	2.368	-0.0120	0.061	0.064
N C	4- 7	2.420	-0.0087	0.063	0.066
N C	4-11	2.511	-0.0164	0.063	0.065
N C	4-18	2.508	-0.0279	0.078	0.081
N C	4-19	2.523	-0.0236	0.078	0.081
N C	4-12	2.877	-0.0202	0.106	0.106
N C	4-15	3.024	-0.0278	0.103	0.103
N C	4-21	3.313	-0.0603	0.136	0.136
N C	4-20	3.439	0.0366	0.115	0.115
N C	4- 8	3.697	-0.0113	0.069	0.069
N C	4-10	3.756	-0.0169	0.069	0.069
N C	4-14	3.782	0.0083	0.202	0.202
N C	4-16	3.919	0.0250	0.198	0.197
N C	4-13	4.079	-0.0410	0.149	0.131
N C	4-17	4.218	-0.0554	0.155	0.138
N C	4- 9	4.220	-0.0154	0.091	0.073
C C	6-10	2.395	-0.0093	0.056	0.059
C C	6- 8	2.396	-0.0098	0.056	0.059
C C	3- 5	2.438	-0.0143	0.056	0.059
C C	3- 6	2.413	0.0098	0.066	0.069
C C	8-10	2.405	-0.0069	0.055	0.058
C C	1- 3	2.443	-0.0115	0.062	0.065
C C	7- 9	2.430	-0.0101	0.055	0.058
C C	9-11	2.431	-0.0092	0.055	0.058
C C	3-21	2.476	-0.0211	0.089	0.092
C C	2- 5	2.511	-0.0213	0.060	0.063
C C	7-11	2.469	-0.0116	0.056	0.059
C C	18-19	2.474	-0.0140	0.078	0.081
C C	7-14	2.492	-0.0300	0.083	0.086
C C	11-16	2.492	-0.0284	0.082	0.085
C C	3-20	2.484	0.0094	0.089	0.092
C C	20-21	2.510	-0.0307	0.084	0.087

(to be contined)

Table S4. *continued*

Type	*At.Num.*	$r_{ij,a}$	$r_{ij,e} - r_{ij,a}$[a]	$u_{ij,exp}$	$u_{ij,h1}$[b]
C C	16-17	2.518	-0.0267	0.082	0.085
C C	10-15	2.506	-0.0078	0.067	0.070
C C	13-14	2.511	-0.0145	0.082	0.085
C C	8-12	2.501	-0.0076	0.067	0.070
C C	7-13	2.532	-0.0200	0.078	0.081
C C	11-17	2.523	-0.0078	0.079	0.082
C C	1-20	2.559	-0.0107	0.082	0.085
C C	1-19	2.570	-0.0214	0.079	0.082
C C	1-18	2.561	-0.0012	0.082	0.085
C C	1-21	2.593	-0.0302	0.084	0.087
C C	6-12	2.579	-0.0258	0.068	0.071
C C	6-15	2.570	-0.0186	0.068	0.071
C C	5- 6	2.637	-0.0401	0.074	0.077
C C	6- 9	2.766	-0.0108	0.067	0.067
C C	7-10	2.807	-0.0100	0.067	0.067
C C	8-11	2.808	-0.0085	0.068	0.067
C C	6-19	3.083	-0.0294	0.128	0.128
C C	8-13	3.016	-0.0037	0.127	0.127
C C	10-17	3.018	0.0178	0.132	0.131
C C	10-16	3.109	-0.0478	0.147	0.147
C C	8-14	3.105	-0.0333	0.147	0.147
C C	6-18	3.279	-0.0966	0.141	0.141
C C	3-11	3.307	-0.0111	0.117	0.117
C C	3- 7	3.180	0.0549	0.121	0.121
C C	7-19	3.350	-0.0745	0.167	0.166
C C	15-18	3.465	-0.0675	0.180	0.180
C C	12-19	3.373	-0.0830	0.189	0.189
C C	3-18	3.391	-0.0147	0.118	0.117
C C	5-21	3.461	-0.0757	0.143	0.142
C C	3-15	3.545	-0.0696	0.170	0.170
C C	2-18	3.435	-0.0299	0.130	0.130
C C	5- 7	3.434	-0.0497	0.111	0.111
C C	11-18	3.615	-0.1312	0.185	0.185
C C	5-11	3.535	-0.0541	0.112	0.112
C C	3-12	3.309	0.0547	0.173	0.173
C C	6-14	3.486	-0.0310	0.154	0.154
C C	6-16	3.479	-0.0114	0.151	0.151
C C	3-19	3.575	-0.0207	0.104	0.104
C C	17-18	3.843	-0.1544	0.241	0.241
C C	13-19	3.644	-0.1314	0.241	0.241
C C	2- 6	3.720	-0.0090	0.069	0.069
C C	5-12	3.664	-0.0332	0.150	0.149
C C	18-21	3.809	-0.1024	0.257	0.257
C C	2-19	3.727	-0.0137	0.099	0.099
C C	5-20	3.700	0.0217	0.103	0.102
C C	6-17	3.708	-0.0153	0.106	0.105
C C	6-13	3.724	-0.0248	0.105	0.105
C C	1- 6	3.775	-0.0177	0.077	0.076
C C	5-15	3.853	-0.0377	0.142	0.142
C C	9-15	3.790	-0.0101	0.073	0.073

(to be contined)

Table S4. *continued*

Type	*At.Num.*	$r_{ij,a}$	$r_{ij,e} - r_{ij,a}$ [a]	$u_{ij,exp}$	$u_{ij,h1}$ [b]
C C	9-12	3.788	-0.0112	0.073	0.072
C C	11-12	3.864	-0.0250	0.074	0.074
C C	7-15	3.857	-0.0213	0.075	0.074
C C	3-16	3.984	0.0111	0.242	0.242
C C	3-14	3.735	0.1050	0.260	0.260
C C	11-19	4.098	-0.0167	0.201	0.183
C C	7-18	4.368	-0.1199	0.199	0.181
C C	9-16	4.323	-0.0464	0.179	0.161
C C	10-12	4.306	-0.0169	0.096	0.078
C C	8-15	4.304	-0.0137	0.096	0.079
C C	15-21	4.701	-0.2378	0.352	0.376
C C	9-14	4.324	-0.0362	0.179	0.161
C C	8-19	4.437	-0.0811	0.243	0.225
C C	1-12	4.264	0.0446	0.245	0.227
C C	2-15	4.639	-0.1282	0.229	0.212
C C	9-13	4.357	0.0002	0.151	0.134
C C	9-17	4.359	0.0216	0.155	0.137
C C	2-12	4.265	0.1056	0.233	0.215
C C	5-13	4.461	-0.0730	0.200	0.182
C C	1- 7	4.451	0.0091	0.153	0.136
C C	5-17	4.668	-0.0902	0.202	0.185
C C	2-11	4.626	-0.0564	0.140	0.123
C C	6-21	4.609	-0.0833	0.191	0.173
C C	3-10	4.542	0.0105	0.139	0.121
C C	2- 7	4.443	0.0571	0.140	0.123
C C	3- 8	4.452	0.0570	0.143	0.126
C C	10-18	4.825	-0.1716	0.266	0.248
C C	11-14	4.601	-0.0358	0.194	0.176
C C	7-16	4.594	-0.0218	0.193	0.175
C C	5- 8	4.674	-0.0652	0.152	0.135
C C	19-20	4.569	0.0634	0.225	0.207
C C	5-10	4.747	-0.0694	0.156	0.138
C C	1-11	4.755	-0.0482	0.122	0.105
C C	1-15	4.890	-0.0716	0.196	0.178
C C	16-18	4.791	0.0013	0.231	0.214
C C	3-17	4.925	-0.1069	0.227	0.209
C C	6-20	4.683	0.0823	0.167	0.149
C C	16-21	4.977	-0.0763	0.352	0.440
C C	15-19	4.795	0.0108	0.203	0.185
C C	14-19	4.795	-0.0583	0.231	0.213
C C	12-20	4.424	0.2905	0.352	0.387
C C	3-13	4.692	0.0283	0.212	0.194
C C	19-21	4.878	-0.0625	0.142	0.125
C C	18-20	4.841	-0.0093	0.135	0.117
C C	5-14	4.840	0.0058	0.232	0.215
C C	8-16	4.916	-0.0346	0.191	0.173
C C	11-21	5.157	-0.1559	0.292	0.274
C C	10-14	4.922	-0.0372	0.191	0.173
C C	12-18	4.955	-0.0833	0.189	0.171
C C	7-17	4.909	-0.0075	0.130	0.112
C C	11-13	4.919	-0.0177	0.130	0.112

(to be contined)

Table S4. *continued*

Type	*At.Num.*	$r_{\mathrm{ij,a}}$	$r_{ij,\mathrm{e}} - r_{ij,\mathrm{a}}$[a]	$u_{ij,\mathrm{exp}}$	$u_{ij,\mathrm{h1}}$[b]
C C	10-19	5.009	-0.0362	0.251	0.233
C C	5-16	5.014	0.0209	0.225	0.207
C C	2-16	5.140	-0.0166	0.311	0.293
C C	2-14	4.745	0.1911	0.339	0.321
C C	3- 9	5.015	0.0379	0.136	0.119
C C	14-20	4.552	0.4037	0.352	0.494
C C	9-19	5.146	-0.0647	0.269	0.251
C C	12-15	5.117	-0.0414	0.113	0.095
C C	5- 9	5.212	-0.0750	0.166	0.148
C C	10-13	5.138	-0.0048	0.142	0.124
C C	8-17	5.135	0.0127	0.143	0.125
C C	1-13	5.118	-0.0009	0.276	0.259
C C	8-18	5.398	-0.1592	0.255	0.237
C C	17-21	5.811	-0.3643	0.352	0.465
C C	7-20	5.040	0.2043	0.281	0.263
C C	1-14	5.163	0.1125	0.332	0.314
C C	9-18	5.585	-0.1835	0.286	0.268
C C	17-19	5.433	-0.0012	0.249	0.232
C C	2-17	5.859	-0.2069	0.301	0.279
C C	1-17	5.843	-0.1477	0.275	0.253
C C	7-21	5.587	-0.0103	0.171	0.149
C C	2-13	5.447	0.0718	0.263	0.245
C C	13-18	5.709	-0.1248	0.252	0.230
C C	1-16	5.786	0.0214	0.276	0.254
C C	11-20	5.754	0.0190	0.162	0.140
C C	15-20	5.951	-0.1069	0.238	0.216
C C	14-15	5.777	-0.0514	0.235	0.213
C C	12-16	5.778	-0.0335	0.232	0.210
C C	12-21	5.644	0.0640	0.212	0.190
C C	1- 8	5.809	-0.0031	0.155	0.133
C C	2-10	5.934	-0.0343	0.136	0.114
C C	2- 8	5.796	0.0515	0.136	0.114
C C	13-20	5.517	0.2759	0.345	0.435
C C	1-10	6.038	-0.0466	0.131	0.109
C C	14-16	6.076	-0.0514	0.337	0.315
C C	16-19	6.067	0.0284	0.238	0.217
C C	14-18	6.149	-0.0476	0.233	0.211
C C	16-20	6.280	-0.0173	0.335	0.314
C C	12-17	6.260	-0.0273	0.144	0.122
C C	13-15	6.268	-0.0307	0.144	0.122
C C	10-21	6.522	-0.1430	0.300	0.279
C C	14-21	6.037	0.1726	0.325	0.304
C C	2- 9	6.432	0.0107	0.117	0.095
C C	1- 9	6.482	-0.0285	0.137	0.115
C C	8-20	6.386	0.2152	0.289	0.267
C C	8-21	6.856	-0.0276	0.192	0.170
C C	17-20	7.268	-0.1837	0.299	0.277
C C	10-20	6.948	0.0708	0.179	0.157
C C	13-21	6.895	0.0297	0.236	0.214
C C	14-17	7.073	-0.0347	0.221	0.199

(to be contined)

Table S4. *continued*

Type	*At.Num.*	$r_{ij,a}$	$r_{ij,e} - r_{ij,a}$ [a]	$u_{ij,exp}$	$u_{ij,h1}$ [b]
C C	13-16	7.078	-0.0271	0.220	0.198
C C	9-21	7.266	-0.0875	0.245	0.223
C C	13-17	7.217	-0.0120	0.200	0.179
C C	9-20	7.229	0.1539	0.228	0.206

[a] Calculated with the DFT- MN15/Def2SVPP cubic force constants (see text).
[b] Calculated with the DFT- MN15/def2TZVP quadratic force constants (see text).

Table S5. Natural Bond Order: (total/covalent/ionic)

Atom			1	2	3	4	5	6	7	8	9
1.	C	t	0.0000	1.0000	0.0000	0.0000	1.0000	0.0000	0.0000	0.0000	0.0000
		c	---	0.9822	0.0000	0.0000	0.9924	0.0000	0.0000	0.0000	0.0000
		i	---	0.0178	0.0000	0.0000	0.0076	0.0000	0.0000	0.0000	0.0000
2.	C	t	1.0000	0.0000	1.0000	0.0000	0.0000	0.0000	0.0000	0.0000	0.0000
		c	0.9822	---	0.9858	0.0000	0.0000	0.0000	0.0000	0.0000	0.0000
		i	0.0178	---	0.0142	0.0000	0.0000	0.0000	0.0000	0.0000	0.0000
3.	C	t	0.0000	1.0000	0.3687	1.6313	0.0000	0.0000	0.0000	0.0000	0.0000
		c	0.0000	0.9858	---	1.0654	0.0000	0.0000	0.0000	0.0000	0.0000
		i	0.0000	0.0142	---	0.5659	0.0000	0.0000	0.0000	0.0000	0.0000
4.	N	t	0.0000	0.0000	1.6313	0.3687	1.0000	1.0000	0.0000	0.0000	0.0000
		c	0.0000	0.0000	1.0654	---	0.7772	0.7985	0.0000	0.0000	0.0000
		i	0.0000	0.0000	0.5659	---	0.2228	0.2015	0.0000	0.0000	0.0000
5.	C	t	1.0000	0.0000	0.0000	1.0000	0.0000	0.0000	0.0000	0.0000	0.0000
		c	0.9924	0.0000	0.0000	0.7772	---	0.0000	0.0000	0.0000	0.0000
		i	0.0076	0.0000	0.0000	0.2228	---	0.0000	0.0000	0.0000	0.0000
6.	C	t	0.0000	0.0000	0.0000	1.0000	0.0000	0.1128	1.2190	0.0000	0.0000
		c	0.0000	0.0000	0.0000	0.7985	0.0000	---	1.1854	0.0000	0.0000
		i	0.0000	0.0000	0.0000	0.2015	0.0000	---	0.0335	0.0000	0.0000
7.	C	t	0.0000	0.0000	0.0000	0.0000	0.0000	1.2190	0.1140	1.5606	0.0000
		c	0.0000	0.0000	0.0000	0.0000	0.0000	1.1854	---	1.5322	0.0000
		i	0.0000	0.0000	0.0000	0.0000	0.0000	0.0335	---	0.0284	0.0000
8.	C	t	0.0000	0.0000	0.0000	0.0000	0.0000	0.0000	1.5606	0.0995	1.2204
		c	0.0000	0.0000	0.0000	0.0000	0.0000	0.0000	1.5322	---	1.2103
		i	0.0000	0.0000	0.0000	0.0000	0.0000	0.0000	0.0284	---	0.0101
9.	C	t	0.0000	0.0000	0.0000	0.0000	0.0000	0.0000	0.0000	1.2204	0.1088
		c	0.0000	0.0000	0.0000	0.0000	0.0000	0.0000	0.0000	1.2103	---
		i	0.0000	0.0000	0.0000	0.0000	0.0000	0.0000	0.0000	0.0101	---
10.	C	t	0.0000	0.0000	0.0000	0.0000	0.0000	0.0000	0.0000	0.0000	1.5580
		c	0.0000	0.0000	0.0000	0.0000	0.0000	0.0000	0.0000	0.0000	1.5359
		i	0.0000	0.0000	0.0000	0.0000	0.0000	0.0000	0.0000	0.0000	0.0221
11.	C	t	0.0000	0.0000	0.0000	0.0000	0.0000	1.5594	0.0000	0.0000	0.0000
		c	0.0000	0.0000	0.0000	0.0000	0.0000	1.4907	0.0000	0.0000	0.0000
		i	0.0000	0.0000	0.0000	0.0000	0.0000	0.0687	0.0000	0.0000	0.0000
12.	C	t	0.0000	0.0000	0.0000	0.0000	0.0000	0.0000	1.0000	0.0000	0.0000
		c	0.0000	0.0000	0.0000	0.0000	0.0000	0.0000	0.9704	0.0000	0.0000
		i	0.0000	0.0000	0.0000	0.0000	0.0000	0.0000	0.0296	0.0000	0.0000

Atom			1	2	3	4	5	6	7	8	9
18.	C	t	0.0000	0.0000	0.0000	0.0000	1.0000	0.0000	0.0000	0.0000	0.0000
		c	0.0000	0.0000	0.0000	0.0000	0.9946	0.0000	0.0000	0.0000	0.0000
		i	0.0000	0.0000	0.0000	0.0000	0.0054	0.0000	0.0000	0.0000	0.0000
19.	C	t	0.0000	0.0000	0.0000	0.0000	1.0000	0.0000	0.0000	0.0000	0.0000
		c	0.0000	0.0000	0.0000	0.0000	0.9974	0.0000	0.0000	0.0000	0.0000
		i	0.0000	0.0000	0.0000	0.0000	0.0026	0.0000	0.0000	0.0000	0.0000
20.	C	t	0.0000	1.0000	0.0000	0.0000	0.0000	0.0000	0.0000	0.0000	0.0000
		c	0.0000	0.9954	0.0000	0.0000	0.0000	0.0000	0.0000	0.0000	0.0000
		i	0.0000	0.0046	0.0000	0.0000	0.0000	0.0000	0.0000	0.0000	0.0000
21.	C	t	0.0000	1.0000	0.0000	0.0000	0.0000	0.0000	0.0000	0.0000	0.0000
		c	0.0000	0.9979	0.0000	0.0000	0.0000	0.0000	0.0000	0.0000	0.0000
		i	0.0000	0.0021	0.0000	0.0000	0.0000	0.0000	0.0000	0.0000	0.0000
22.	Cu	t	0.0000	0.0000	1.0000	0.0000	0.0000	0.0000	0.0000	0.0000	0.0000
		c	0.0000	0.0000	0.3081	0.0000	0.0000	0.0000	0.0000	0.0000	0.0000
		i	0.0000	0.0000	0.6919	0.0000	0.0000	0.0000	0.0000	0.0000	0.0000

Atom			10	11	12	13	14	15	16	17	18
5.	C	t	0.0000	0.0000	0.0000	0.0000	0.0000	0.0000	0.0000	0.0000	1.0000
		c	0.0000	0.0000	0.0000	0.0000	0.0000	0.0000	0.0000	0.0000	0.9946
		i	0.0000	0.0000	0.0000	0.0000	0.0000	0.0000	0.0000	0.0000	0.0054
6.	C	t	0.0000	1.5594	0.0000	0.0000	0.0000	0.0000	0.0000	0.0000	0.0000
		c	0.0000	1.4907	0.0000	0.0000	0.0000	0.0000	0.0000	0.0000	0.0000
		i	0.0000	0.0687	0.0000	0.0000	0.0000	0.0000	0.0000	0.0000	0.0000
7.	C	t	0.0000	0.0000	1.0000	0.0000	0.0000	0.0000	0.0000	0.0000	0.0000
		c	0.0000	0.0000	0.9704	0.0000	0.0000	0.0000	0.0000	0.0000	0.0000
		i	0.0000	0.0000	0.0296	0.0000	0.0000	0.0000	0.0000	0.0000	0.0000
8.	C	t	0.0000	0.0000	0.0000	0.0000	0.0000	0.0000	0.0000	0.0000	0.0000
		c	0.0000	0.0000	0.0000	0.0000	0.0000	0.0000	0.0000	0.0000	0.0000
		i	0.0000	0.0000	0.0000	0.0000	0.0000	0.0000	0.0000	0.0000	0.0000
9.	C	t	1.5580	0.0000	0.0000	0.0000	0.0000	0.0000	0.0000	0.0000	0.0000
		c	1.5359	0.0000	0.0000	0.0000	0.0000	0.0000	0.0000	0.0000	0.0000
		i	0.0221	0.0000	0.0000	0.0000	0.0000	0.0000	0.0000	0.0000	0.0000
10.	C	t	0.1064	1.2216	0.0000	0.0000	0.0000	0.0000	0.0000	0.0000	0.0000
		c	---	1.2105	0.0000	0.0000	0.0000	0.0000	0.0000	0.0000	0.0000
		i	---	0.0111	0.0000	0.0000	0.0000	0.0000	0.0000	0.0000	0.0000
11.	C	t	1.2216	0.1194	0.0000	0.0000	0.0000	1.0000	0.0000	0.0000	0.0000
		c	1.2105	---	0.0000	0.0000	0.0000	0.9700	0.0000	0.0000	0.0000
		i	0.0111	---	0.0000	0.0000	0.0000	0.0300	0.0000	0.0000	0.0000
12.	C	t	0.0000	0.0000	0.0000	1.0000	1.0000	0.0000	0.0000	0.0000	0.0000
		c	0.0000	0.0000	---	0.9920	0.9930	0.0000	0.0000	0.0000	0.0000
		i	0.0000	0.0000	---	0.0080	0.0070	0.0000	0.0000	0.0000	0.0000
13.	C	t	0.0000	0.0000	1.0000	0.0000	0.0000	0.0000	0.0000	0.0000	0.0000
		c	0.0000	0.0000	0.9920	---	0.0000	0.0000	0.0000	0.0000	0.0000
		i	0.0000	0.0000	0.0080	---	0.0000	0.0000	0.0000	0.0000	0.0000
14.	C	t	0.0000	0.0000	1.0000	0.0000	0.0000	0.0000	0.0000	0.0000	0.0000
		c	0.0000	0.0000	0.9930	0.0000	---	0.0000	0.0000	0.0000	0.0000
		i	0.0000	0.0000	0.0070	0.0000	---	0.0000	0.0000	0.0000	0.0000

Atom		10	11	12	13	14	15	16	17	18
15. C	t	0.0000	1.0000	0.0000	0.0000	0.0000	0.0000	1.0000	1.0000	0.0000
	c	0.0000	0.9700	0.0000	0.0000	0.0000	---	0.9930	0.9920	0.0000
	i	0.0000	0.0300	0.0000	0.0000	0.0000	---	0.0070	0.0080	0.0000
16. C	t	0.0000	0.0000	0.0000	0.0000	0.0000	1.0000	0.0000	0.0000	0.0000
	c	0.0000	0.0000	0.0000	0.0000	0.0000	0.9930	---	0.0000	0.0000
	i	0.0000	0.0000	0.0000	0.0000	0.0000	0.0070	---	0.0000	0.0000
17. C	t	0.0000	0.0000	0.0000	0.0000	0.0000	1.0000	0.0000	0.0000	0.0000
	c	0.0000	0.0000	0.0000	0.0000	0.0000	0.9920	0.0000	---	0.0000
	i	0.0000	0.0000	0.0000	0.0000	0.0000	0.0080	0.0000	---	0.0000

Atom		19	20	21	22	23	24	25	26	27
1. C	t	0.0000	0.0000	0.0000	0.0000	0.0000	0.0000	0.0000	0.0000	0.0000
	c	0.0000	0.0000	0.0000	0.0000	0.0000	0.0000	0.0000	0.0000	0.0000
	i	0.0000	0.0000	0.0000	0.0000	0.0000	0.0000	0.0000	0.0000	0.0000
2. C	t	0.0000	1.0000	1.0000	0.0000	0.0000	0.0000	0.0000	0.0000	0.0000
	c	0.0000	0.9954	0.9979	0.0000	0.0000	0.0000	0.0000	0.0000	0.0000
	i	0.0000	0.0046	0.0021	0.0000	0.0000	0.0000	0.0000	0.0000	0.0000
3. C	t	0.0000	0.0000	0.0000	1.0000	0.0000	0.0000	0.0000	0.0000	0.0000
	c	0.0000	0.0000	0.0000	0.3081	0.0000	0.0000	0.0000	0.0000	0.0000
	i	0.0000	0.0000	0.0000	0.6919	0.0000	0.0000	0.0000	0.0000	0.0000
22. Cu	t	0.0000	0.0000	0.0000	4.6313	0.3390	0.0000	0.0000	0.0000	0.0000
	c	0.0000	0.0000	0.0000	---	0.0651	0.0000	0.0000	0.0000	0.0000
	i	0.0000	0.0000	0.0000	---	0.2740	0.0000	0.0000	0.0000	0.0000
23. Cl	t	0.0000	0.0000	0.0000	0.3390	3.6610	0.0000	0.0000	0.0000	0.0000
	c	0.0000	0.0000	0.0000	0.0651	---	0.0000	0.0000	0.0000	0.0000
	i	0.0000	0.0000	0.0000	0.2740	---	0.0000	0.0000	0.0000	0.0000

Table S6. Total scattering intensities and background lines for (**1**) molecule

Curve LD

s	Total Int	Background	s	Total Int	Background
4.20	0.28005	0.29433	10.20	0.24678	0.24465
4.40	0.28390	0.29827	10.40	0.24441	0.24238
4.60	0.28741	0.30201	10.60	0.24148	0.24011
4.80	0.29474	0.30533	10.80	0.23809	0.23790
5.00	0.30789	0.30797	11.00	0.23440	0.23577
5.20	0.32395	0.30959	11.20	0.23094	0.23377
5.40	0.33363	0.30987	11.40	0.22779	0.23191
5.60	0.33415	0.30876	11.60	0.22560	0.23023
5.80	0.32711	0.30639	11.80	0.22451	0.22873
6.00	0.31742	0.30302	12.00	0.22428	0.22735
6.20	0.30647	0.29895	12.20	0.22413	0.22606
6.40	0.29553	0.29453	12.40	0.22415	0.22481
6.60	0.28556	0.29010	12.60	0.22416	0.22356
6.80	0.27784	0.28593	12.80	0.22439	0.22228
7.00	0.27359	0.28216	13.00	0.22441	0.22094
7.20	0.27220	0.27883	13.20	0.22377	0.21950
7.40	0.27151	0.27590	13.40	0.22248	0.21798
7.60	0.27074	0.27331	13.60	0.22023	0.21640
7.80	0.26965	0.27097	13.80	0.21744	0.21484
8.00	0.26819	0.26876	14.00	0.21431	0.21336
8.20	0.26599	0.26660	14.20	0.21150	0.21203
8.40	0.26399	0.26444	14.40	0.20929	0.21090
8.60	0.26262	0.26227	14.60	0.20791	0.20998
8.80	0.26148	0.26008	14.80	0.20757	0.20925
9.00	0.25993	0.25786	15.00	0.20762	0.20862
9.20	0.25747	0.25563	15.20	0.20742	0.20804
9.40	0.25457	0.25342	15.40	0.20707	0.20746
9.60	0.25191	0.25125	15.60	0.20645	0.20687
9.80	0.25013	0.24908	15.80	0.20572	0.20630
10.00	0.24860	0.24689	16.00	0.20526	0.20574

Curve SD

s	Total Int	Background	s	Total Int	Background
13.00	1.02780	1.01634	22.40	1.05040	1.05025
13.20	1.03120	1.01626	22.60	1.05070	1.05113
13.40	1.03170	1.01620	22.80	1.05110	1.05200
13.60	1.02910	1.01618	23.00	1.05160	1.05286
13.80	1.02480	1.01625	23.20	1.05220	1.05372
14.00	1.01990	1.01644	23.40	1.05310	1.05458
14.20	1.01560	1.01670	23.60	1.05430	1.05544
14.40	1.01280	1.01695	23.80	1.05550	1.05631
14.60	1.01170	1.01714	24.00	1.05690	1.05718
14.80	1.01170	1.01725	24.20	1.05830	1.05804
15.00	1.01240	1.01737	24.40	1.05960	1.05891
15.20	1.01360	1.01761	24.60	1.06100	1.05978
15.40	1.01510	1.01806	24.80	1.06220	1.06065
15.60	1.01660	1.01873	25.00	1.06330	1.06152
15.80	1.01810	1.01958	25.20	1.06410	1.06239
16.00	1.01920	1.02048	25.40	1.06480	1.06327
16.20	1.01990	1.02134	25.60	1.06520	1.06415
16.40	1.02010	1.02212	25.80	1.06550	1.06505
16.60	1.01990	1.02287	26.00	1.06570	1.06596

16.80	1.02000	1.02365	26.20	1.06610	1.06689
17.00	1.02090	1.02455	26.40	1.06660	1.06786
17.20	1.02300	1.02558	26.60	1.06740	1.06885
17.40	1.02580	1.02669	26.80	1.06840	1.06987
17.60	1.02890	1.02782	27.00	1.06960	1.07093
17.80	1.03210	1.02891	27.20	1.07100	1.07202
18.00	1.03500	1.02992	27.40	1.07250	1.07314
18.20	1.03730	1.03086	27.60	1.07390	1.07428
18.40	1.03860	1.03173	27.80	1.07540	1.07547
18.60	1.03880	1.03258	28.00	1.07680	1.07669
18.80	1.03790	1.03342	28.20	1.07820	1.07796
19.00	1.03650	1.03428	28.40	1.07970	1.07927
19.20	1.03490	1.03515	28.60	1.08110	1.08063
19.40	1.03370	1.03603	28.80	1.08260	1.08204
19.60	1.03320	1.03693	29.00	1.08400	1.08347
19.80	1.03330	1.03783	29.20	1.08550	1.08491
20.00	1.03410	1.03875	29.40	1.08700	1.08634
20.20	1.03560	1.03970	29.60	1.08840	1.08773
20.40	1.03780	1.04069	29.80	1.08940	1.08907
20.60	1.04040	1.04172	30.00	1.09060	1.09038
20.80	1.04280	1.04276	30.20	1.09170	1.09166
21.00	1.04500	1.04378	30.40	1.09290	1.09290
21.20	1.04660	1.04477	30.60	1.09410	1.09412
21.40	1.04760	1.04572	30.80	1.09520	1.09529
21.60	1.04830	1.04664	31.00	1.09630	1.09643
21.80	1.04900	1.04755	31.20	1.09730	1.09752
22.00	1.04960	1.04846	31.40	1.09820	1.09858
22.20	1.05010	1.04936	31.60	1.09880	1.09961
13.00	1.02780	1.01634	22.40	1.05040	1.05025

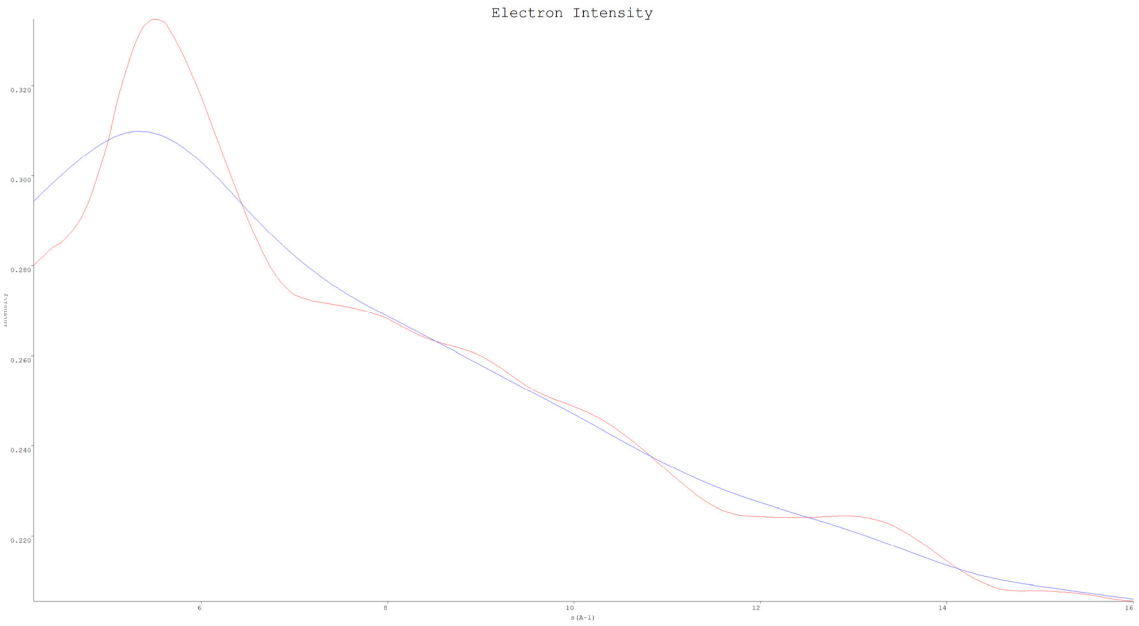

Figure S1. Total experimental scattering intensity curve and background line for(**1**), (LD)

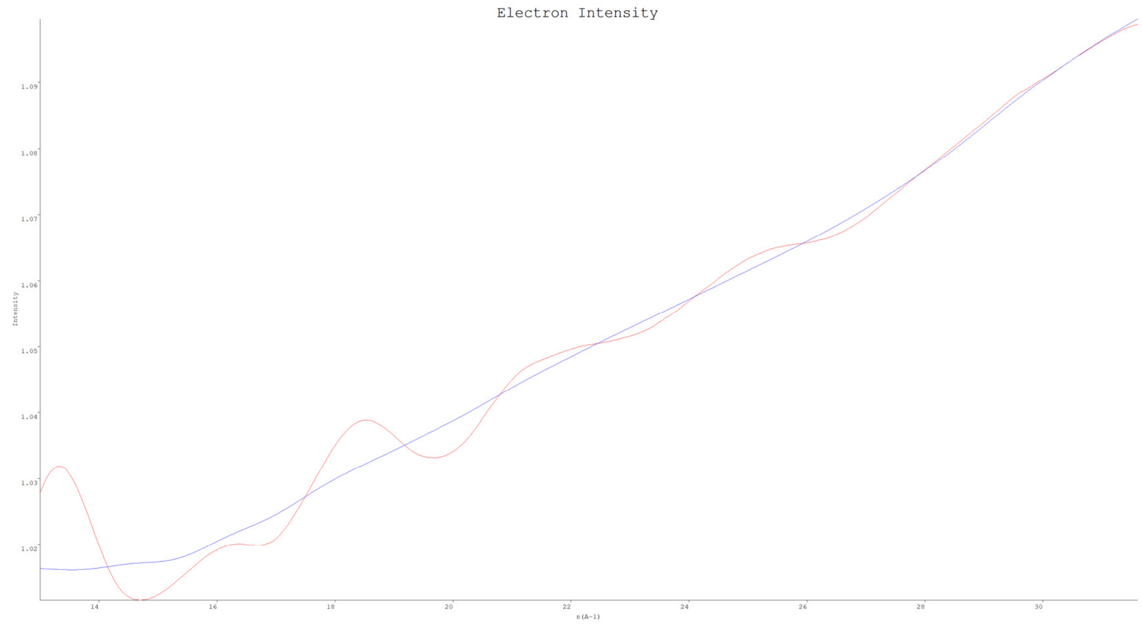

Figure S2. Total experimental scattering intensity curve and background line for(**1**), (SD)
